# Intrathoracic hemorrhage by deep pericardial sutures as detected by transesophageal echocardiography: report of two cases

**DOI:** 10.1186/s40981-019-0285-3

**Published:** 2019-10-25

**Authors:** Mirei Nagai, Satoshi Kurokawa, Makoto Ozaki, Minoru Nomura

**Affiliations:** Department of Anesthesiology, Tokyo Women’s Medical University, 8-1 Kawada-cho, Shinjuku, Tokyo, 162-8666 Japan

**Keywords:** Off-pump coronary artery bypass grafting, Deep pericardial suture, Transesophageal echocardiography, Hemorrhagic complication

## Abstract

**Background:**

The use of deep pericardial suture (DPS) is a widely used technique to lift the heart and expose the targeted vessels during off-pump coronary artery bypass grafting (OPCAB). Several reports alert massive bleeding due to DPS, especially for the patients with perioperative administration of tissue plasminogen activator, low molecular weight heparin, and administration of double antiplatelet agents.

**Case presentation:**

We report two cases of bleeding followed by huge hematoma formation in the left thoracic cavity caused by DPS during OPCAB. In one, bleeding was caused by damage to the left lower pulmonary vein and damage to the diaphragmatic artery in the other. Transesophageal echocardiography (TEE) is a potent tool for detecting complications and identifying the location of bleeding.

**Conclusions:**

TEE is useful for finding hemorrhagic complications and for determining the points of bleeding based on the location of the hematoma.

## Background

Exposure of target coronary arteries is essential for successful off-pump coronary artery bypass grafting (OPCAB). The use of deep pericardial suture (DPS) is a common technique to lift the heart and expose the targeted vessels during OPCAB [1]. This procedure holds the advantage of not requiring special devices and incurring extra costs [2], whereas there have been several reports on complications of bleeding due to DPS [3–6].

Herein, we report two OPCAB cases with three-vessel disease in which transesophageal echocardiography (TEE) was important for detecting the bleeding caused by DPS.

Written consents were obtained from both patients prior to the writing of this report.

## Case presentation

### Case 1

An 83-year-old male with a history of myocardial infarction was scheduled to undergo OPCAB. His medical history included chronic kidney disease, paroxysmal atrial fibrillation, type ΙΙ diabetes mellitus (DM), and dyslipidemia. He took low-dose oral aspirin (100 mg/day) until the day of the surgery because of his history of myocardial infarction.

The left internal thoracic artery was initially anastomosed to the left anterior descending artery (LAD) using gauze pads under the left ventricle and application of a tissue stabilizer (Octopus™, Medtronic Inc., Minneapolis, MN). After completion of LAD anastomosis, two DPSs were placed in the posterior pericardium between the inferior vena cava and the left lower pulmonary vein (LLPV), exposing the lateral to inferior walls. The saphenous vein graft was sequentially anastomosed to the obtuse marginal and the posterior descending arteries with the application of tissue stabilizer. Flowmetry showed both grafts provided excellent flow to the arterial territories. At the end of the OPCAB, his hemodynamic parameters were stable, and surgical bleeding was well controlled. In addition, TEE showed good cardiac contraction and no hematoma in either the pericardial or the intrathoracic spaces.

**Fig. 1:**
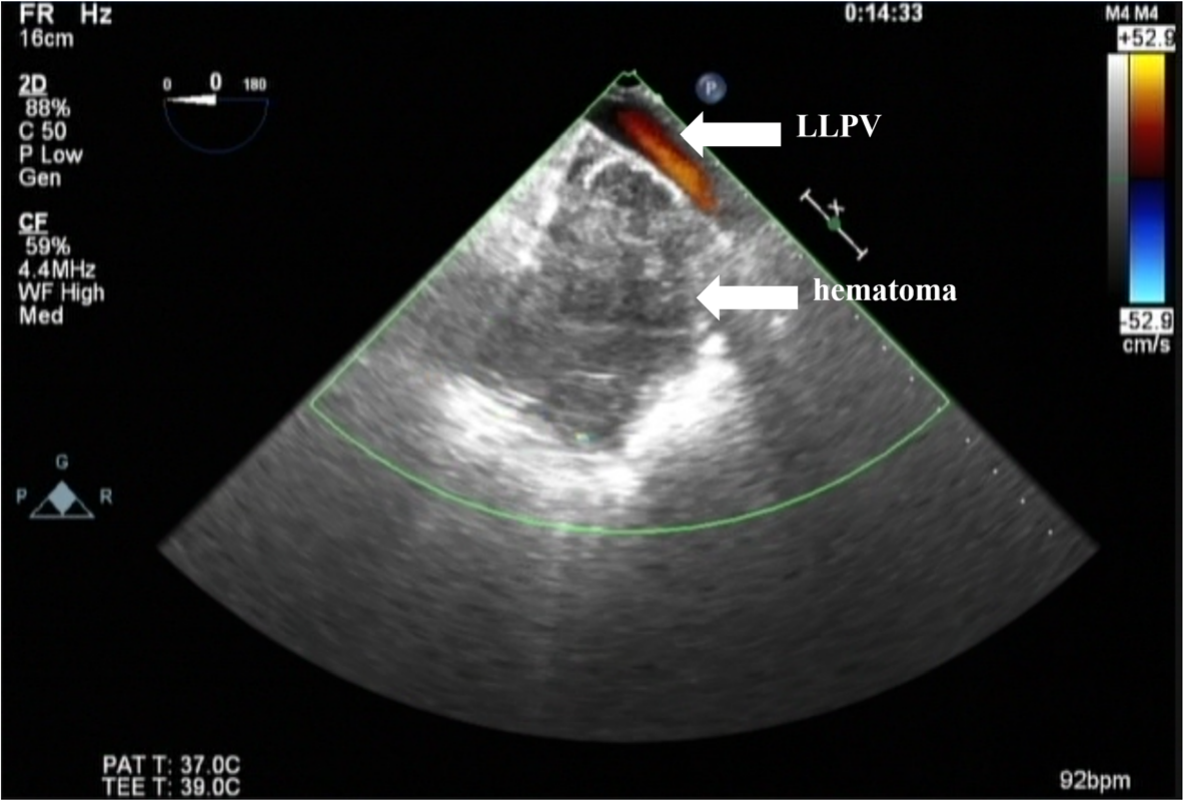
Left-sided intrathoracic hematoma adjacent to the left lower pulmonary vein area. Left lower pulmonary vein flow is depicted using the color Doppler imaging mode. LLPV, left lower pulmonary vein

The patient was extubated 21 h after the surgery in the intensive care unit. About an hour after extubation, he lost consciousness and exhibited ventricular tachycardia. He was soon resuscitated with cardioversion and amiodarone infusion. A diagnosis of cardiac tamponade was made based on transthoracic echocardiogram findings although bleeding from mediastinal and pericardial drainage tubes was < 10 mL/h for 12 h, and he was taken to the operation theater for emergency re-exploration. Intraoperative TEE revealed a small amount of pericardial effusion; however, there is a huge hematoma in the left thoracic cavity, including an area just adjacent to the LLPV (Fig. 1). Surgical inspection supported that the location of the hematoma was consistent with TEE findings. His hemodynamics stabilized after removal of the hematoma. The location of hematoma implied bleeding from the LLPV due to DPSs, leading to a huge hematoma. His postoperative course was uneventful.

### Case 2

A 58-year-old male with unstable angina was scheduled to have OPCAB under the support of intra-aortic balloon pumping (IABP). Preoperative coronary risk factors were type-ΙΙ DM and hypertension. He was given heparin intravenously to control the activated partial thrombin time in the range of 40–50 s after IABP insertion. Heparin infusion was stopped 3 h before the OPCAB.

**Fig. 2:**
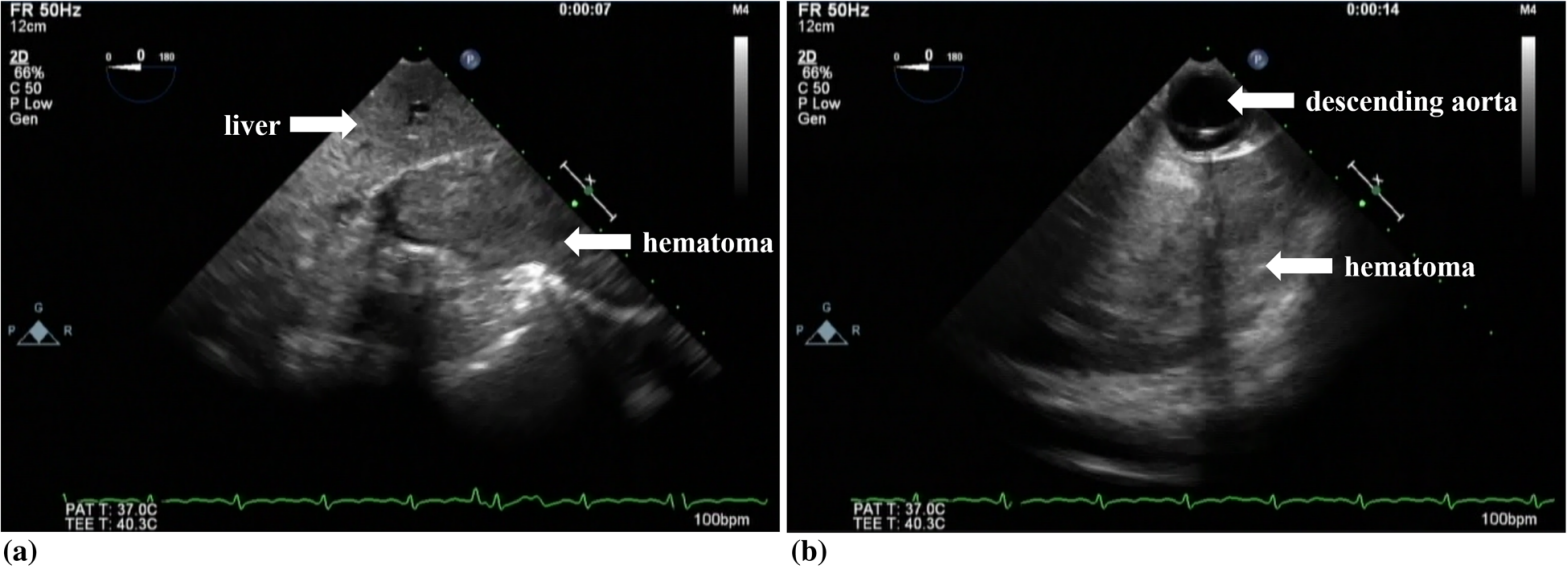
Hematoma in the left thoracic cavity at the level of the diaphragm. Hematoma is observed at the level of the liver (**a**). A giant hematoma is visualized in the left thoracic cavity behind the descending aorta (**b**)

Using the same techniques as the first case to expose the targeted vessels, the right internal thoracic artery was anastomosed to the LAD by placing gauze pads and applying a tissue stabilizer. Then, a combination of two DPSs and tissue stabilizer enabled anastomoses of the left internal thoracic artery to the posterolateral artery and the gastroepiploic artery to the posterior descending artery. After anastomosing all grafts to the targeted coronary arteries, flowmetry confirmed good flowrates and profiles. However, the patient’s hemodynamics was still unstable after chest closure. TEE exploration revealed a massive hematoma in the left thoracic cavity, especially near the diaphragm (Fig. 2). The left thoracic cavity was exposed, and the hematoma was manually removed. Careful surgical inspection found the bleeding from the artery on the surface of the diaphragm. Surgical ligation of the artery was accomplished. The patient’s condition stabilized without IABP, and he was extubated 19 h after the surgery. He had an uneventful postoperative course.

## Discussion

Local stabilization of cardiac wall motion is critically important for achieving accurate anastomoses [1]. The heart can be appropriately positioned by pulling on multiple or single DPS, placed in the posterior pericardium [1, 2]. During DPS stitching, great caution should be taken to avoid serious bleeding. Although this complication seems rare because, to our best knowledge, only four case reports have been published [3–6], DPS may cause life-threatening bleeding. In one out of the four published cases, the patient died because of aortic injury [3]. In the remaining three cases, all authors suggested the preoperative administration of tissue plasminogen activator, the postoperative administration of low molecular weight heparin, and the postoperative administration of double antiplatelet agents presumably increased the bleeding [4–6]. In contrast, in our two cases, no agents relating to coagulation cascade or platelet function, except a low dose of aspirin or preoperative heparin infusion, were administered. When we detected bleeding, activated clotting time, platelet count, and fibrinogen level were 104 s, 8.2 × 10^4^/μL, and 242 mg/dL in case 1, and 110 s, 8.0 × 10^4^/μL, and 359 mg/dL in case 2, respectively. The present cases suggest that DPS can cause serious bleeding complication, even in the absence of aggressive medication for fibrinolysis, anticoagulation, or antiplatelet aggregation.

Zamvar et al. mentioned that sufficient deflation of the lung before DPS created more room for surgeons to avoid the lung injuries [4]. While complete deflation of the lung was performed in these two cases to prevent injury to organs or tissues adjacent to the pericardium, injuries could not be avoided. Based on this, we have to keep in mind that lung deflation is not a fail-safe means of preventing organ or tissue injuries.

TEE was a useful tool for detecting bleeding complications in the thorax, pericardium, and para-aortic space [4–6]. Intrathoracic fluid or blood accumulation is often encountered during cardiac surgery and is easily visualized using TEE with the counterclockwise rotation of the probe from the mid-esophageal four-chamber view, considering the left-sided accumulation in a patient in a supine position. Typically, fluid or blood accumulates in the dorsal and caudal portion of the pleural space, i.e., at the bottom of the left thorax beside the descending aorta [7]. In contrast to this, hematomas due to vessel injury by DPS are localized at the bottom of the left-sided thorax as well as, to a certain extent, in the field adjacent to the injury site. Therefore, presence of the latter is typical of this DPS complication. Indeed, we detected numerous hematomas, including a giant one at the bottom of the thorax in both cases. The area surrounding the LLPV immediately behind the pericardium in case 1 and the wide area along with the diaphragm in case 2 are notable examples for the localization of the injury site. The previous reports identified the aorta, pulmonary veins, esophagus, and lung as organs susceptible to injuries by DPS [3–6]. Our case 2 suggested that the diaphragm also requires attention during DPS stitching.

## Conclusions

In summary, our cases demonstrated that, first, a massive bleeding due to DPS could occur in the absence of aggressive anticoagulant or antiplatelet therapies, and second, arteries on the diaphragm were one of the injured vessels. In addition, TEE is a potent tool for detecting complications and identifying the location of bleeding.

## Data Availability

The data used during the current study are available from the corresponding author on reasonable request.

## Abbreviations

DM: Diabetes mellitus
DPS: Deep pericardial suture
IABP: Intra-aortic balloon pumping
LAD: Left anterior descending artery
LLPV: Left lower pulmonary vein
OPCAB: Off-pump coronary artery bypass grafting
TEE: Transesophageal echocardiography
